# Civic participation among young people in Chile: an association analysis in times of COVID-19

**DOI:** 10.1590/1980-220X-REEUSP-2024-0372en

**Published:** 2025-07-28

**Authors:** María Angélica Saldías-Fernández, Lylian Macias-Inzunza, Mauricio Arias-Rojas, Edgardo Stanic-Bustamante, Denisse Parra-Giordano

**Affiliations:** 1 Universidad de Chile, Facultad de Medicina, Programa Doctorado en Salud Pública, Santiago, Chile.; 2 Universidad de Santiago de Chile, Escuela de Enfermería, Santiago, Chile.; 3 Universidad de Antioquia, Facultad de Enfermería, Medellín, Colombia.; 4 Centro de Salud Familiar Dr. Félix de Amesti, Santiago, Chile.; 5 Universidad de Chile, Facultad de Medicina, Departamento de Enfermería, Santiago, Chile.

**Keywords:** Young Adult, Adolescent, Health Promotion, Disease Prevention, Community Participation, Health Policy, Adulto Jovem, Adolescente, Promoção da Saúde, Prevenção de Doenças, Participação da Comunidade, Política de Saúde

## Abstract

**Objective:**

To analyze the association between sociodemographic and socio-health factors on the civic participation of young people in Chile.

**Method:**

A cross-sectional study using the Tenth National Youth Survey. Descriptive statistics and models were used to examine associations between sociodemographic, socio-health, social participation, and sociopolitical variables in Chile.

**Results:**

Female sex and education levels indicated greater political participation. Being single, not being heads of household, being happy, having a positive outlook on their future, being satisfied with their finances, health, leisure time, having time available to spend with friends and family, and the country’s characteristic democracy showed significant associations. No identification with specific political sectors, interest in political matters, and self-identification as not actively participating in politics had significant associations.

**Conclusion:**

Young people are essential for building future citizenship. Their unique characteristics must be strengthened through effective citizen participation strategies, mediated by education that fosters awareness of their own value as individuals who impact public policy.

## RESUMO

**Objetivo:**

Analisar a associação entre fatores sociodemográficos e sociossanitários na participação cívica de jovens no Chile.

**Método:**

Estudo transversal utilizando a Décima Pesquisa Nacional de Juventude. Estatísticas descritivas e modelos foram usados ​​para examinar associações entre variáveis sociodemográficas, sociosanitárias, de participação social e sociopolíticas no Chile.

**Resultados:**

Sexo feminino e escolaridade indicam maior participação política. Solteiros, não chefes de família, felizes, com uma perspectiva positiva sobre o próprio futuro, satisfeitos com suas finanças, saúde, tempo de lazer, tempo disponível para passar com amigos e familiares e com a democracia que caracteriza o país apresentaram associações significativas, sem qualquer identificação com setores políticos específicos, interesses em assuntos políticos, autodefinição como não participante ativo na política, com associações significativas.

**Conclusão:**

Os jovens são essenciais para a construção da cidadania futura. As características individuais devem ser fortalecidas por meio de estratégias efetivas de participação cidadã, mediadas por uma educação que permita que os cidadãos tomem consciência do seu próprio valor como indivíduos que impactam as políticas públicas.

## INTRODUCTION

Citizen participation (CP) and community involvement are a fundamental principle in healthcare, oriented towards social justice and a means to positively impact people’s lives^(1,2)^. Public healthcare emerges as a fundamental pillar in democratic societies^(2,3,4,5)^, especially in the sphere of public policies (PP), allowing citizens to directly influence the decisions that affect their lives. In the health sector, where universal health coverage is advancing towards global consolidation, public healthcare takes on special relevance in defining public priorities. The search for fair criteria for the distribution of healthcare in the face of scarce resources has generated intense debate in the fields of health, bioethics and philosophy, highlighting the urgent need for an inclusive approach and the promotion of participatory strategies aligned with the population’s values and preferences^(6)^.

The incorporation of CP into health policymaking and health technology assessment has proven indispensable for the design of informed and responsible health technologies. CP focuses on capturing a diversity of viewpoints, extending beyond the actors traditionally involved in the process. Thus, CP draws on methods such as in-person debates and digital forums that foster constructive dialogue among different participants, in which citizens have the opportunity to consider the social and ethical implications inherent in health technologies^(7)^.

There is widespread recognition that the participation of service users and their caregivers in health system planning and policy processes can strengthen health systems. However, most of these patients come from high-income countries^(2,3,6)^, and there is little evidence of the relationship between perceived causes and solutions to health inequalities among citizens^(5)^.

It is critical to keep in mind that CP is also impacted by people’s health, limiting their ability to act^(3)^. In this regard, nursing plays a fundamental role in CP. For this development, the discipline proposes six patterns of knowledge, emphasizing the emancipatory pattern, in which Chinn and Kramer recognize the importance of social justice in nursing as a science with a critical awareness of the social and political status quo^(8)^.

In this way, CP promotes an active and empowered citizenry to have a responsible and receptive participation in PP in health^(3)^. Decision-makers should consider strategies that encourage different methods of CP, with multiple forms of expression of ideas^(5,9)^, and inform and listen to the population, using the feedback obtained in decision-making^(3)^. Local residents are the ones who become leading figures of collective well-being^(10)^.

In Chile, CP has been recognized through Law 20,500, also known as the Law of Associations and Citizen Participation in Public Management^(11)^. This law emphasizes the importance of actively involving citizens, especially young people (YP), in deliberation and decision-making.

Research has shown that by working with communities, it is possible to develop services that utilize assets and are meaningful to the local population^(12)^.

In this regard, a first step towards identifying aspects related to youth CP is the National Youth Survey, which seeks to obtain relevant and timely information for diagnosing the realities in Chile. The 2022 Tenth National Youth Survey^(13)^ was characterized by confinement due to COVID-19. In this context, public space is understood beyond the physical, fitting into a logic of production of social integration and CP, with its development being crucial^(10)^.

Seen in these terms, the present research process aims to analyze the association of sociodemographic factors, aspects related to life and health, on CP of YP in Chile, with the purpose of contributing with evidence that allows a redefinition of public priorities that guide the development of participatory humanitarian environments, health strategies related to health promotion and disease prevention in Chile focused on YP, in addition to representing an input in terms of professional training that emphasizes the importance of CP in care decisions of people and their environment.

## METHOD

### Study Design

This is a cross-sectional design study, with a descriptive and inferential scope based on data from the 2022 Tenth National Youth Survey^(13)^, which has anonymized and freely accessible data.

### Survey Overview

Chile conducted the 2022 Tenth National Youth Survey with a random sample of YP between 15–29 years old from all socioeconomic groups and living in all regions, with a margin of error of + or − 1% for the total results with maximum variance and 95% confidence.

### Data Collection and Sample

Data were collected from December 2021 to May 2022 through in-person, in-home surveys of YP aged 15 to 29 using tablets, lasting an average of 40 minutes. Stratified sampling was used proportionally for each of the participants’ regions of residence in Chile. A total of 9,700 YP were surveyed.

### Data Analysis

All measures were summarized using means, standard deviations, and ranges for continuous measures, and frequencies and percentages for categorical measures. Sociodemographic characteristics were analyzed. Association analyses were performed using the chi-square (X^2^) test and Fisher’s exact test for categorical variables. After identifying associations, a logistic regression analysis was performed to observe the prediction of sociodemographic variables on CP. However, no statistically significant models were found. All analyses were completed using the Statistical Package for the Social Sciences version 26^(14)^.

## RESULTS

A summary of the study population characteristics is presented in Tables 1, 2, and 3. The majority of YP between 15 and 29 years of age in the 2022 Tenth National Youth Survey^(13)^ were women, living in urban areas, with a medium level of education completed in public schools, single, without employment activity, and economically dependent on others. No political sector is recognized, nor do they show interest in participating in national politics. Furthermore, they are mostly happy, satisfied with life and the economy. Concerning health, leisure time, and interactions with family, they perceive themselves as very satisfied. They are not mostly satisfied with democracy, and their view of Chile in 5 years does not seem to be different from the current one. In relation to active CP in the last 12 months, around 53% claim to have done so as well as active participation at the unrecognized leadership level and in PP development processes.

**Table 1:** Participant sociodemographic characteristics – Santiago, Chile 2025.

Variable	Categories	Frequency (N = 9,700)	Percentage (%)
Sex	Male	4,335	44.7
	Female	5,365	55.3
Age	Mean (SD)	22	4.46
Origin	Rural	1,014	10.5
	Urban	8,686	89.5
Level of education	Basic education	345	3.6
	Middle education	3,360	34.6
	Professional-technician	1,496	15.4
	Vocational education	1,601	16.5
	Higher education	2,898	29.9
Type of graduating school	Municipal	5,951	61.4
	Paid private	596	6.1
	Subsidized private	3,153	32.5
Marital status	In a relationship	146	1.5
	Living with a partner	1,599	16.5
	Other	44	0.5
	Single	7,911	81.6
Occupation	Formal employment	3,354	34.6
	Not working	4,811	49.6
	Informal employment	1,535	15.8
Head of household	No	7,779	80.2
	Yes	1,921	19.8
Political sector of identification	Center	1,047	10.8
	Center-right	402	4.1
	Center-left	778	8
	Right	696	7.2
	Left	998	10.3
	None of these positions	5,779	59.6
Considers that there is equality of positions in politics according to gender	Agree	1,616	16.7
	Disagree	149	1.5
	Strongly agree	7,294	75.2
	Strongly disagree	151	1.6
	Neither agree nor disagree	490	5.1
Level of interest in politics	Interested	1,930	19.9
	Very interested	701	7.2
	Not at all interested	3,829	39.5
	Somewhat interested	3,240	33.4
Visualizing their own lives in 5 years	Same as now	899	9.3
	Better than now	8,624	88.9
	Worse than now	177	1.8

Source: Own elaboration, based on the 2022 Tenth National Youth Survey^(13)^.

**Table 2:** Socio-health variables and aspects related to life – Santiago, Chile 2025.

Variable	Categories	Frequency (N = 9,700)	Percentage (%)
Level of happiness	Happy	5,118	52.8
	Very happy	2,503	25.8
	Not at all happy	67	0.7
	Neither happy nor unhappy	1,757	18.1
	Not very happy	255	2.6
Level of life satisfaction	Dissatisfied	270	2.8
	Very dissatisfied	83	0.9
	Very satisfied	3,378	34.8
	Neither satisfied nor dissatisfied	1,833	18.9
	Satisfied	4,136	42.6
Level of financial satisfaction	Dissatisfied	835	8.6
	Very dissatisfied	393	4.1
	Very satisfied	2,303	23.7
	Neither satisfied nor dissatisfied	2,732	28.2
	Satisfied	3,437	35.4
Level of health satisfaction	Dissatisfied	463	4.8
	Very dissatisfied	158	1.6
	Very satisfied	4,368	45
	Neither satisfied nor dissatisfied	1,487	15.3
	Satisfied	3,224	33.2
Level of leisure time satisfaction	Dissatisfied	1,218	12.6
	Very dissatisfied	646	6.7
	Very satisfied	2,790	28.8
	Neither satisfied nor dissatisfied	2,444	25.2
	Satisfied	2,602	26.8
Level of satisfaction with family relationships	Dissatisfied	260	2.7
	Very dissatisfied	123	1.3
	Very satisfied	5,479	56.5
	Neither satisfied nor dissatisfied	1,062	10.9
	Satisfied	2,776	28.6
Level of satisfaction with democracy	Dissatisfied	1,864	19.2
	Very dissatisfied	1,044	10.8
	Very satisfied	422	4.4
	Neither satisfied nor dissatisfied	4,554	46.9
	Satisfied	1,816	18.7
Visualization of Chile’s perception in 5 years	Same as now	3,532	36.4
	Better than now	3,523	36.3
	Worse than now	2,645	27.3

Source: Own elaboration, based on the 2022 Tenth National Youth Survey^(13)^.

**Table 3:** Participant citizen participation – Santiago, Chile 2025.

Variable	Categories	Frequency (N = 9,700)	Percentage (%)
Active political participation in the last 12 months	No	4,603	47.5
Political participation as a leader in the last 12 months	Yes	5,097	52.5
Participation in community service or volunteering	No	8,278	85.3
	Yes	1,422	14.7
Active political participation in the last 12 months	No	6,281	64.8
Political participation as a leader in the last 12 months	Yes	3,419	35.2
Participation in community service or volunteering	No	9,136	94.2
	Yes	564	5.8

Source: Own elaboration, based on the 2022 Tenth National Youth Survey^(13)^.

The analysis of the associations between sociodemographic characteristics and participation is shown in Table 4.

**Table 4:** Associations between sociodemographic characteristics and active political participation in the last 12 months – Santiago, Chile 2025.

Variable	Categories	Active political participation in the last 12 months		p-value
		Yes	No	
Sex	Female	3,021 (56.3)	2,344 (43.70)	0.000
	Male	1,582 (36.5)	2,753 (63.5)	
Level of education	Basic education	153 (44.3)	192 (55.7)	0.000
	Middle education	1,760 (52.4)	1,600 (47.6)	
	Professional-technician	781 (52.2)	715 (47.8)	
	Vocational education	777 (48.5)	824 (51.5)	
	Higher education	1,626 (56.1)	1,272 (43.9)	
Type of graduating school	Paid private	295 (49.5)	301 (50.5)	0.306
	Subsidized private	1,664 (52.8)	1,489 (47.2)	
	Municipal	3,138 (52.7)	2,813 (47.3)	
Marital status	Single	4,257 (53.8)	3,654 (46.2)	0.000
	In a relationship	82 (56.2)	64 (43.8)	
	Living with a partner	730 (45.7)	869 (54.3)	
	Other	28 (63.6)	16 (36.4)	
Occupation	Not working	2,560 (53.2)	2,251 (46.8)	0.240
	Informal employment	814 (53)	721 (47.0)	
	Formal employment	1,723 (51.4)	1,631 (48.6)	
Head of household	Yes	928 (48.3)	993 (51.7)	0.000
	No	4,169 (53.6)	3,610 (46.4)	
Political sector	Right	378 (54.3)	318 (45.7)	0.000
	Center-right	246 (61.2)	156 (38.8)	
	Center	576 (55)	471 (45.0)	
	Center-left	467 (60)	311 (40)	
	Left	588 (58.9)	410 (41.1)	
	None of these positions	2,842 (49.2)	2,937 (50.8)	
Considers that there is equality of positions in politics according to gender	Strongly disagree	62 (41.1)	89 (58.9)	0.000
	Disagree	62 (41.6)	87 (58.4)	
	Neither agree nor disagree	207 (42.2)	283 (57.8)	
	Agree	796 (49.3)	820 (50.7)	
	Strongly agree	3,970 (54.4)	3,324 (45.6)	
Level of interest in politics	Not at all interested	1,698 (44.3)	2,131 (55.7)	0.000
	Slightly interested	1,739 (53.7)	1,501 (46.3)	
	Interested	1,170 (60.6)	760 (39.4)	
	Very interested	490 (69.9)	211 (30.1)	
Visualizing their own lives in 5 years	Better than now	4,592 (53.2)	4,032 (46.8)	0.000
	Same as now	411 (45.7)	488 (54.3)	
	Worse than now	94 (53.1)	83 (46.9)	
Level of happiness	Not at all happy	31 (46.3)	36 (53.7)	0.053
	Not very happy	137 (53.7)	118 (46.3)	
	Neither happy nor unhappy	977 (55.6)	780 (44.4)	
	Happy	2,660 (52)	2,458 (48.0)	
	Very happy	1,292 (51.6)	1,211 (48.4)	
Level of satisfaction with life	Very dissatisfied	42 (50.6)	41 (49.4)	0.448
	Dissatisfied	142 (52.6)	128 (47.4)	
	Neither satisfied nor dissatisfied	987 (53.8)	846 (46.2)	
	Satisfied	2,192 (53.0)	1,944 (47.0)	
	Very satisfied	1,734 (51.3)	1,644 (48.7)	
Level of satisfaction with the economy	Very dissatisfied	196 (49.9)	197 (50.1)	0.051
	Dissatisfied	445 (53.3)	390 (46.7)	
	Neither satisfied nor dissatisfied	1,384 (50.7)	1,348 (49.3)	
	Satisfied	1,813 (52.7)	1,624 (47.3)	
	Very satisfied	1,259 (54.7)	1,044 (45.3)	
Level of satisfaction with health	Very dissatisfied	96 (60.8)	62 (39.2)	0.000
	Dissatisfied	261 (56.4)	202 (43.6)	
	Neither satisfied nor dissatisfied	732 (49.2)	755 (50.8)	
	Satisfied	1,622 (50.3)	1,602 (49.7)	
	Very satisfied	2,386 (54.6)	1,982 (45.4)	
Level of satisfaction with rest time	Very dissatisfied	366 (56.7)	280 (43.3)	0.013
	Dissatisfied	651 (53.4)	567 (46.6)	
	Neither satisfied nor dissatisfied	1,260 (51.6)	1,184 (48.4)	
	Satisfied	1,313 (50.5)	1,289 (49.5)	
	Very satisfied	1,507 (54.0)	1,283 (46.0)	
Level of satisfaction with family relationships	Very dissatisfied	71 (57.7)	52 (42.3)	0.017
	Dissatisfied	161 (61.9)	99 (38.1)	
	Neither satisfied nor dissatisfied	562 (52.9)	500 (47.1)	
	Satisfied	1,427 (51.4)	1,349 (48.6)	
	Very satisfied	2,876 (52.5)	2,603 (47.5)	
Level of satisfaction with democracy	Very dissatisfied	500 (47.9)	544 (52.1)	0.023
	Dissatisfied	1,002 (53.8)	862 (46.2)	
	Neither satisfied nor dissatisfied	2,393 (52.5)	2,161 (47.5)	
	Satisfied	975 (53.7)	841 (46.3)	
	Very satisfied	227 (53.8)	195 (46.2)	
Visualization of Chile’s perception in 5 years	Better than now	1,856 (52.7)	1,667 (47.3)	0.570
	Same as now	1,833 (51.9)	1,699 (48.1)	
	Worse than now	1,408 (53.2)	1,237 (46.8)	

Source: Own elaboration, based on the 2022 Tenth National Youth Survey^(13)^.

Under the assumption of normality, significant associations can be identified between the study population characteristics and active political participation in the past 12 months. Females were the most prominent political participants, compared to males, with considerable significance (p-value = 0.00). Those who self-report average levels of education report significant political participation (p-value = 0.00) in other types of education achieved in the age group. Single YP (p-value = 0.00), not heads of household (p-value = 0.00), self-defined as happy (p-value = 0.053), with a positive view of their own life in the future (p-value = 0.00), satisfied with the current economy (p-value = 0.051), with health (p-value = 0.00), with time to rest (p-value = 0.013), with time available to share with friends and family (p-value = 0.017) and with the country’s democracy (p-value = 0.023) showed significant associations. And YP without any identification in specific political sectors (p-value = 0.00) or with interests in political matters (p-value = 0.00) define themselves as not active participants in politics, with significant associations.

## DISCUSSION

This research process identified certain associations between sociodemographic factors and aspects related to life and health as well as CP of YP in Chile. This is significant because understanding age-related expectations for community participation can help policymakers design and adapt appropriate services.

The results show a level of interest in politics of 27.1%, which is positive considering that when people engage as public representatives, they are able to raise collective concerns^(7,10)^ on diverse subjects, with health being of great interest. This is demonstrated by the fact that 88.9% see their lives improving in five years. In the same context, it is suggested that CP is necessary to achieve a distribution of political power, achieve community power to impact PP and achieve social change^(15)^. They developed a CP ladder, where with the implementation of long-term community control mechanisms and investment of resources, citizens will be able to carry out CP. They also introduced the idea of private financing for this type of process. This is critical because the level of satisfaction with the country’s democracy is significantly associated with active participation in the past 12 months.

The level of satisfaction with health stands out, with 78.2% very satisfied or satisfied in the context of COVID-19, where a period of confinement was experienced in the country, showing 52% with a very good or good perception of healthcare services^(10)^. A study in Argentina suggests that the non- in-person modality provided peace of mind in preserving people’s health^(10)^.

It is complex, since almost half of people (46.9%) do not usually respond about their level of satisfaction with democracy. According to a study conducted in Canada, people need health policies to consider public opinion^(16)^. Thus, in Colombia, 89% would approve of government management if it developed social cohesion policies^(17)^.

In addition, the literature emphasizes that greater public participation in political processes is expected, as involvement could foster agreement on priorities in decision-making. The results show that only 5.8% of YP are willing to participate in the development of PP for youth, consistent with research in Colombia that mentions an antipathy toward political issue^(17)^. The low levels of political participation over the past 12 months could be related to the COVID-19 lockdown, due to the isolation itself and the lack of prioritization of needs. This pandemic process can be linked to an increase in participation in community service or volunteering over the past 12 months, compared to other participations, with an increase in participation in neighborhood councils, soup kitchens, among others^(10)^.

Hence, 56.5% of survey participants are very satisfied with their family relationships, according to a study conducted in Colombia, which shows that people interact most with their family (77.3%). Furthermore, this is the group with the greatest relationship (88.6%). At the collective level, 73.5% declare interest in participating in their localities^(17)^. This point is fundamental, as research conducted in the United Kingdom and South Africa highlights the importance of mutual benefit in achieving CP. In teams where there was no vision for collaborative work, with decisions depending on a single person, the team disintegrated without achieving participation. The former emphasizes CP through publicly funded research, and the latter emphasizes a social justice approach with results that benefit the community^(1)^.

This research showed a significant association between CP and the level of satisfaction with health, as well as a study in Sweden, where participation is impacted by good healthcare^(2)^.

Health promotion and disease prevention are significant when they are intended to be enhanced through health education, since with educated populations it is possible to strengthen CP in care decisions^(18)^. This is supported by the fact that 89% of the population would like to be trained in topics related to politics, as well as participate in meetings with government authorities, in order to propose solutions and not just obtain information about the problems that afflict them^(17)^.

Likewise, this research supports that active political participation in the last 12 months has a significant association with the lack of identification in political sectors, interests in the subject matter or not self-defining as an active participant in politics. Communication strategies, such as communication campaigns framed in a balance between effectiveness and ethics^(19)^, represent public health approaches.

In this context, in Chile, recent socio-political and health tensions due to the COVID-19 pandemic may have generated some stress and fatigue among citizens^(20)^. However, CP strategies such as promoting spaces for constructive dialogue allow differences to be addressed productively and agreements to be reached that are acceptable to the majority of the community^(21,22)^.

YP show a significant association between available time to share with friends and family and CP; thus, CP can impact health promotion and prevention. A study in Mexico argues that close social ties and family can enhance and strengthen individuals’ cohesion-participation with their environment^(17)^, resulting in healthier biopsychosocial behaviors^(23,24,25)^. In YP, it is significant, since the development of a substantial perspective of citizenship can favor the incorporation of YP into public life as long as a social, cultural and institutional context is built based on meaningful participation and dialogue with youth^(26)^.

The level of satisfaction with democracy is significantly associated with political participation over the past twelve months. In Colombia, 56% of respondents rate the government 6/10^(17)^. Taking ownership of the right to participate is recognized as one of the essential practices for being an active citizen, where they must be previously informed and present belonging to the community^(26)^. Thus, the COVID-19 lockdown allowed us to perceive the environment differently, creating stronger ties with the neighborhood^(10)^. Thus, it should be kept in mind that people’s perception of their sense of social belonging to their family and place of origin impacts their civic and electoral development^(17)^.

It is recognized that the first step to being an active citizen is being informed^(26)^, but to achieve higher levels of CP it is also necessary to inform and listen to stakeholders and use people’s feedback in decision-making. Similarly, this study in the US suggests that CP is a source of improvement in the face of regulatory violations, and suggests that CP can positively impact administration, quality of care, people’s rights, among others, highlighting the value of participation in areas such as health, which are highly professionalized, where there are a large number of regulations and training^(3)^.

The need for people’s participation in the policies that guide social services is also highlighted, although the path to implementing these policies in PP is difficult. Staff approaches to user participation are crucial for people’s opportunities to participate in decision-making, particularly in the case of those with extensive care needs and limited autonomy^(2)^.

CP has significance in political models^(22)^, as it emphasizes the real needs of people, who, in alliance with other actors, can influence the agenda and participate in their own solutions. It is essential that the government acts as a moderator in decision-making and controls political power^(27,28,29)^. Thus, CP and culture generate a strong social impact^(26)^.

Moreover, CP research is key to the co-production and development of PP^(3,4)^. As opportunities for public participation in health research increase, greater participation is anticipated^(16)^. In this regard, public participation should be considered a requirement for obtaining health funding in various institutions^(30)^.

Finally, the concept of CP has been impacted by pressures to be used as a symbol for groups that prioritize inclusive and diverse CP^(4)^. As a limitation, this study only shows one version of the survey. Other research suggests comparing it with non- pandemic periods to identify the sequence of CP evolution over time in YP.

## CONCLUSION

This study allowed us to analyze the association of sociodemographic factors, life-related aspects, and health with CP of YP in Chile. Females describe greater political participation, and educational levels indicate significant political participation. Single YP, not heads of household, who self-identified as happy, with a positive outlook on their own future, satisfied with the current economy, health, leisure time, and time available to spend with friends and family, and with the democracy that characterizes the country demonstrated significant associations. Likewise, YP without any identification with specific political sectors or interests in political matters define themselves as not active participants in politics, with meaningful associations. YP are essential for building future citizenship. In this regard, their unique characteristics must be strengthened through effective CP strategies, mediated by education that allows them to recognize their own value as people who impact PP.

## Data Availability

The entire dataset supporting the results of this study is available at the Digital Documentation Center of the National Youth Institute of the Government of Chile at the URL https://www.injuv.gob.cl/encuestanacionaldejuventud in November 2022.
