# Supplementary material for: Civic participation among young people in Chile: an association analysis in times of COVID-19
Source: Rev Esc Enferm USP. 2025 Jul 28;59:e20240372. doi: 10.1590/1980-220X-REEUSP-2024-0372en (PMC12313322; doi:10.1590/1980-220X-REEUSP-2024-0372en)
Supplement: Translation

# Translation: Participación ciudadana en personas jóvenes en Chile: un análisis de asociación en tiempos de COVID-19

**DOI:** 10.1590/1980-220X-REEUSP-2024-0372es

**Authors:** María Angélica Saldías-Fernández, Lylian Macias-Inzunza, Mauricio Arias-Rojas, Edgardo Stanic-Bustamante, Denisse Parra-Giordano

**Affiliations:** 1 Universidad de Chile, Facultad de Medicina, Programa Doctorado en Salud Pública, Santiago, Chile.; 2 Universidad de Santiago de Chile, Escuela de Enfermería, Santiago, Chile.; 3 Universidad de Antioquia, Facultad de Enfermería, Medellín, Colombia.; 4 Centro de Salud Familiar Dr. Félix de Amesti, Santiago, Chile.; 5 Universidad de Chile, Facultad de Medicina, Departamento de Enfermería, Santiago, Chile.

**Keywords:** Adulto Joven, Adolescente, Promoción de la Salud, Prevención de Enfermedades, Participación de la Comunidad, Política de Salud

## Abstract

**Objetivo:**

Analizar la asociación entre factores sociodemográficos y sociosanitarios sobre la participación ciudadana de personas jóvenes en Chile.

**Método:**

Estudio transversal utilizando la Décima Encuesta Nacional de Juventudes. Se utilizaron estadística descriptiva y modelos para examinar asociaciones entre variables sociodemográficas, sociosanitarias, de participación social y sociopolítica en Chile.

**Resultados:**

Sexo femenino y escolaridad refieren mayor participación política. Soltería, no jefes de hogar, felices, mirada positiva propia vida en el futuro, satisfechos con economía, salud, tiempo de descanso, tiempo disponible para compartir con amigos y familia y democracia que caracteriza el país demostraron asociaciones significativas, sin identificación alguna en sectores políticos determinados, intereses en materias políticas, autodefinición como no participante activo en política, con asociaciones significativas.

**Conclusión:**

Personas jóvenes son fundamentales para construir ciudadanía futura. Se deben fortalecer características propias mediante estrategias de participación ciudadana efectivas, mediadas por educación que permitan ser conscientes del propio valor como personas que impactan en política pública.

## INTRODUCCIÓN

La participación ciudadana (PC) y el involucramiento de la comunidad son un principio fundamental en la atención de salud, orientados a la justicia social y un medio para impactar positivamente en la vida de las personas^(1,2)^. La PC emerge como un pilar cardinal en las sociedades democráticas^(2–5)^, especialmente en la esfera de las políticas públicas (PP), que permite a los ciudadanos influir de manera directa en las decisiones que inciden en sus vidas. En el ámbito de la salud, en el cual la cobertura sanitaria universal avanza hacia su consolidación global, la PC cobra especial relevancia en la definición de prioridades públicas. La búsqueda de criterios justos para la distribución de atención sanitaria frente a la escasez de recursos ha generado un intenso debate en los campos de la salud, la bioética y la filosofía, subrayando la imperiosa necesidad de un enfoque inclusivo y de promoción de estrategias participativas alineadas con los valores y preferencias de la población^(6)^.

La incorporación de la PC en la formulación de políticas de salud y en la evaluación de tecnologías sanitarias ha demostrado ser indispensable para el diseño de PP informadas y responsables. La PC se centra en capturar una diversidad de puntos de vista, extendiéndose más allá de los actores tradicionalmente involucrados en el proceso. Así, la PC se nutre de métodos como debates presenciales y foros digitales que fomentan el diálogo constructivo entre los distintos participantes, en el que los ciudadanos tienen la oportunidad de considerar las implicaciones sociales y éticas inherentes a las tecnologías sanitarias^(7)^.

Existe un amplio reconocimiento que la participación de usuarios de servicios y sus cuidadores en los procesos de planificación y políticas del sistema de salud puede fortalecer los sistemas de salud. Sin embargo, la mayoría proviene de países de altos ingresos^(2,3,6)^, y se presenta poca evidencia entre la relación de las causas percibidas y las soluciones a las desigualdades en salud entre la ciudadanía^(5)^.

Es crítico tener presente que la PC también está impactada por la salud de las personas, delimitando su posibilidad de actuación^(3)^. En tal sentido, enfermería tiene un rol fundamental desde la PC. Para este desarrollo, la disciplina plantea seis patrones del conocimiento, dando énfasis al patrón emancipatorio, en el que Chinn y Kramer reconocen la importancia de la justicia social en el quehacer de enfermería como una ciencia con una conciencia crítica sobre el *statu quo* social y político^(8)^.

De esta forma, la PC promueve una ciudadanía activa y empoderada para tener una participación responsable y receptiva en PP en salud^(3)^. Los tomadores de decisión deben considerar estrategias que fomenten diferentes métodos de PC, con múltiples formas de expresión de ideas^(5,9)^, e informar y escuchar a la población, debiendo utilizar las retroalimentaciones obtenidas en la toma de decisiones^(3)^. Los residentes locales son quienes se convierten en protagonistas del bienestar colectivo^(10)^.

En Chile, la PC ha sido reconocida a través de la Ley n^o^ 20.500, también conocida como Ley de Asociaciones y Participación Ciudadana en la Gestión Pública^(11)^. Esta ley enfatiza la importancia de involucrar activamente a la ciudadanía, especialmente a los jóvenes, en la deliberación y toma de decisiones.

Las investigaciones han demostrado que trabajando con las comunidades es posible desarrollar servicios que utilicen activos y sean significativo para la población local^(12)^.

En ello, un primer paso para la identificación de aspectos relativos a PC de jóvenes es la Encuesta Nacional de Juventudes, que busca obtener información pertinente y oportuna para el diagnóstico de las realidades en Chile. La Décima Encuesta Nacional de Juventudes 2022^(13)^ estuvo caracterizada por el confinamiento producto del COVID-19. En este contexto, espacio público se entiende más allá de lo físico, encajándose en una lógica de producción de integración social y de PC, siendo crucial su desarrollo^(10)^.

Mirado así, el presente proceso investigativo tiene por objetivo analizar la asociación de factores sociodemográficos, aspectos relacionados con la vida y la salud, sobre la PC de personas jóvenes (PJ) en Chile, con el propósito de contribuir con evidencia que permita una redefinición de prioridades públicas que orienten el desarrollo de entornos humanitarios participativos, estrategias sanitarias relacionadas con la promoción de salud y prevención de enfermedades en Chile enfocadas en PJ, además de representar un insumo en materia de formación profesional que enfatice la importancia de la PC en las decisiones de cuidado de las personas y su entorno.

## MÉTODO

### Tipo de Estudio

Estudio de diseño transversal, con alcance descriptivo e inferencial a partir de los datos de la Décima Encuesta Nacional de Juventudes 2022^(13)^, que dispone de datos anonimizados y de libre acceso.

### Descripción General de la Encuesta

Chile realizó la Décima Encuesta Nacional de Juventudes 2022 con una muestra aleatoria de PJ entre 15–29 años de todos los grupos socioeconómicos y que vivieran en todas las regiones, con un margen de error de +o- 1% para los resultados totales con varianza máxima y 95% de confianza.

### Recolección de Datos Y Muestra

Los datos fueron recolectados de diciembre del 2021 a mayo del 2022, a través de encuestas presenciales en el hogar de jóvenes que tuvieran entre 15 y 29 años, utilizando *tablets*, con una duración de 40 minutos en promedio. Se usó un muestreo estratificado proporcional para cada una de las regiones de residencia de los participantes en Chile. En total 9.700 jóvenes fueron encuestados.

### Análisis de Datos

Todas las medidas se resumieron utilizando medias, desviaciones estándar y rangos, para las medidas continuas, y frecuencias y porcentajes, para las medidas categóricas. Se analizaron las características sociodemográficas. Se realizaron análisis de asociación utilizando la prueba de chi-cuadrado (X^2^) y la prueba exacta de Fisher para variables categóricas. Posterior a la identificación de asociaciones se realizó un análisis de regresión logística para observar la predicción de las variables sociodemográficas sobre la PC. Sin embargo, no se encontraron modelos que fueran estadísticamente significativos. Todo el análisis se completó empleando el *software Statistical Package for the Social Sciences*, versión 26^(14)^.

## RESULTADOS

Se presenta un resumen de las características de la población estudiada (Tablas 1, 2 y 3). La mayoría de las PJ entre 15 y 29 años en la Décima Encuesta Nacional de Juventudes 2022^(13)^ fueron mujeres, viviendo en zonas urbanas, con nivel medio de escolaridad cursado en colegios públicos, solteras, sin actividad laboral y dependientes económicamente de otros. Ningún sector político es reconocido, así como tampoco se muestran interesados en participar en la política nacional. En adición, se muestran mayoritariamente felices, satisfechos con la vida y economía. A propósito de la salud, el tiempo de descanso y las interacciones con familiares, se autoperciben como muy satisfechos. No se presentan mayormente satisfechos con la democracia, y su visualización de Chile a 5 años no pareciera ser diferente a la actual. Respecto a la PC activa de los últimos 12 meses, alrededor de un 53% afirma haberlo hecho, del mismo modo la participación activa a nivel dirigencial no reconocido y en procesos de elaboración de PP.

**Tabla 1:** Características sociodemográficas de los participantes – Santiago, Chile 2025.

Variable	Categorías	Frecuencia (N = 9.700)	Porcentaje (%)
Sexo	Hombre	4.335	44,7
	Mujer	5.365	55,3
Edad	Media (DE)	22	4,46
Procedencia	Rural	1.014	10,5
	Urbano	8.686	89,5
Nivel escolar	Básica	345	3,6
	Media	3.360	34,6
	Media técnica	1.496	15,4
	Técnica superior	1.601	16,5
	Universitaria	2.898	29,9
Tipo de colegio de egreso	Municipal	5.951	61,4
	Particular pagado	596	6,1
	Particular subvencionado	3.153	32,5
Estado civil	Comprometido	146	1,5
	Convive con pareja	1.599	16,5
	Otro	44	0,5
	Soltero	7.911	81,6
Ocupación	Empleo formal	3.354	34,6
	No trabaja	4.811	49,6
	Trabajo informal	1.535	15,8
Jefe de hogar	No	7.779	80,2
	Sí	1.921	19,8
Sector político de identificación	Centro	1.047	10,8
	Centro derecha	402	4,1
	Centro izquierda	778	8
	Derecha	696	7,2
	Izquierda	998	10,3
	Ninguna de esas posiciones	5.779	59,6
Considera que sí existe igualdad de cargos en política de acuerdo con el género	De acuerdo	1.616	16,7
	En desacuerdo	149	1,5
	Muy de acuerdo	7.294	75,2
	Muy en desacuerdo	151	1,6
	Ni de acuerdo, ni en desacuerdo	490	5,1
Nivel de interés en política	Interesado	1.930	19,9
	Muy interesado	701	7,2
	Nada interesado	3.829	39,5
	Poco interesado	3.240	33,4
Visualización de la propia vida en 5 años	Igual que ahora	899	9,3
	Mejor que ahora	8.624	88,9
	Peor que ahora	177	1,8

Fuente: Elaboración propia, a partir de Décima Encuesta Nacional de Juventudes 2022^(13)^.

**Tabla 2:** Variables sociosanitarias y aspectos relacionados con la vida – Santiago, Chile 2025.

Variable	Categorías	Frecuencia (N = 9.700)	Porcentaje (%)
Nivel de felicidad	Feliz	5.118	52,8
	Muy feliz	2.503	25,8
	Nada feliz	67	0,7
	Ni feliz, ni infeliz	1.757	18,1
	No muy feliz	255	2,6
Nivel de satisfacción con la vida	Insatisfecho	270	2,8
	Muy insatisfecho	83	0,9
	Muy satisfecho	3.378	34,8
	Ni satisfecho, ni insatisfecho	1.833	18,9
	Satisfecho	4.136	42,6
Nivel de satisfacción con la economía	Insatisfecho	835	8,6
	Muy insatisfecho	393	4,1
	Muy satisfecho	2.303	23,7
	Ni satisfecho, ni insatisfecho	2.732	28,2
	Satisfecho	3.437	35,4
Nivel de satisfacción con la salud	Insatisfecho	463	4,8
	Muy insatisfecho	158	1,6
	Muy satisfecho	4.368	45
	Ni satisfecho, ni insatisfecho	1.487	15,3
	Satisfecho	3.224	33,2
Nivel de satisfacción con el tiempo de descanso	Insatisfecho	1.218	12,6
	Muy insatisfecho	646	6,7
	Muy satisfecho	2.790	28,8
	Ni satisfecho, ni insatisfecho	2.444	25,2
	Satisfecho	2.602	26,8
Nivel de satisfacción con relaciones familiares	Insatisfecho	260	2,7
	Muy insatisfecho	123	1,3
	Muy satisfecho	5.479	56,5
	Ni satisfecho, ni insatisfecho	1.062	10,9
	Satisfecho	2.776	28,6
Nivel de satisfacción con la democracia	Insatisfecho	1.864	19,2
	Muy insatisfecho	1.044	10,8
	Muy satisfecho	422	4,4
	Ni satisfecho, ni insatisfecho	4.554	46,9
	Satisfecho	1.816	18,7
Visualización de la percepción de Chile en 5 años	Igual que ahora	3.532	36,4
	Mejor que ahora	3.523	36,3
	Peor que ahora	2.645	27,3

Fuente: Elaboración propia, a partir Décima Encuesta Nacional de Juventudes 2022^(13)^.

**Tabla 3:** Participación ciudadana de los participantes – Santiago, Chile 2025.

Variable	Categorías	Frecuencia (N = 9700)	Porcentaje (%)
Participación política activa en los últimos 12 meses	No	4.603	47,5
	Sí	5.097	52,5
Participación política como dirigente en los últimos 12 meses	No	8.278	85,3
	Sí	1.422	14,7
Participación en ayuda a la comunidad o voluntariado	No	6.281	64,8
	Sí	3.419	35,2
Participación en elaboración de políticas para la juventud	No	9.136	94,2
	Sí	564	5,8

Fuente: Elaboración propia, a partir de Décima Encuesta Nacional de Juventudes 2022^(13)^.

El análisis de las asociaciones entre las características sociodemográficas y la participación se muestra en la Tabla 4.

**Tabla 4:** Asociaciones entre características sociodemográficas y participación política activa en los últimos 12 meses – Santiago, Chile 2025.

Variable	Categorías	Participación política activa en los últimos 12 meses		Valor de p
		Si	No	
Sexo	Mujer	3.021 (56,3)	2.344 (43,70)	0,000
	Hombre	1.582 (36,5)	2.753 (63,5)	
Nivel escolar	Básica	153 (44,3)	192 (55,7)	0,000
	Media	1.760 (52,4)	1.600 (47,6)	
	Media técnica	781 (52,2)	715 (47,8)	
	Técnica superior	777 (48,5)	824 (51,5)	
	Universitaria	1.626 (56,1)	1.272 (43,9)	
Tipo de colegio de egreso	Particular pagado	295 (49,5)	301 (50,5)	0,306
	Particular subvencionado	1.664 (52,8)	1.489 (47,2)	
	Municipal	3.138 (52,7)	2.813 (47,3)	
Estado civil	Soltero	4.257 (53,8)	3.654 (46,2)	0,000
	Comprometido	82 (56,2)	64 (43,8)	
	Convive con pareja	730 (45,7)	869 (54,3)	
	Otro	28 (63,6)	16 (36,4)	
Ocupación	No trabaja	2.560 (53,2)	2.251 (46,8)	0,240
	Trabajo informal	814 (53)	721 (47,0)	
	Empleo formal	1.723 (51,4)	1.631 (48,6)	
Jefe de hogar	Sí	928 (48,3)	993 (51,7)	0,000
	No	4.169 (53,6)	3.610 (46,4)	
Sector político	Derecha	378 (54,3)	318 (45,7)	0,000
	Centro derecha	246 (61,2)	156 (38,8)	
	Centro	576 (55)	471 (45,0)	
	Centro izquierda	467 (60)	311 (40)	
	Izquierda	588 (58,9)	410 (41,1)	
	Ninguna de esas posiciones	2.842 (49,2)	2.937 (50,8)	
Considera que sí existe igualdad de cargos en política de acuerdo con el género	Muy en desacuerdo	62 (41,1)	89 (58,9)	0,000
	En desacuerdo	62 (41,6)	87 (58,4)	
	Ni en acuerdo, ni desacuerdo	207 (42,2)	283 (57,8)	
	De acuerdo	796 (49,3)	820 (50,7)	
	Muy de acuerdo	3.970 (54,4)	3.324 (45,6)	
Nivel de interés en política	Nada interesado	1.698 (44,3)	2.131 (55,7)	0,000
	Poco interesado	1.739 (53,7)	1.501 (46,3)	
	Interesado	1.170 (60,6)	760 (39,4)	
	Muy interesado	490 (69,9)	211 (30,1)	
Visualización de la propia vida en 5 años	Mejor que ahora	4.592 (53,2)	4.032 (46,8)	0,000
	Igual que ahora	411 (45,7)	488 (54,3)	
	Peor que ahora	94 (53,1)	83 (46,9)	
Nivel de felicidad	Nada feliz	31 (46,3)	36 (53,7)	0,053
	No muy feliz	137 (53,7)	118 (46,3)	
	Ni feliz, ni infeliz	977 (55,6)	780 (44,4)	
	Feliz	2.660 (52)	2.458 (48,0)	
	Muy feliz	1.292 (51,6)	1.211 (48,4)	
Nivel de satisfacción con la vida	Muy insatisfecho	42 (50,6)	41 (49,4)	0,448
	Insatisfecho	142 (52,6)	128 (47,4)	
	Ni satisfecho, ni insatisfecho	987 (53,8)	846 (46,2)	
	Satisfecho	2.192 (53,0)	1.944 (47,0)	
	Muy satisfecho	1.734 (51,3)	1.644 (48,7)	
Nivel de satisfacción con la economía	Muy insatisfecho	196 (49,9)	197 (50,1)	0,051
	Insatisfecho	445 (53,3)	390 (46,7)	
	Ni satisfecho, ni insatisfecho	1.384 (50,7)	1.348 (49,3)	
	Satisfecho	1.813 (52,7)	1.624 (47,3)	
	Muy satisfecho	1.259 (54,7)	1.044 (45,3)	
Nivel de satisfacción con la salud	Muy insatisfecho	96 (60,8)	62 (39,2)	0,000
	Insatisfecho	261 (56,4)	202 (43,6)	
	Ni satisfecho, ni insatisfecho	732 (49,2)	755 (50,8)	
	Satisfecho	1.622 (50,3)	1.602 (49,7)	
	Muy satisfecho	2.386 (54,6)	1.982 (45,4)	
Nivel de satisfacción con el tiempo de descanso	Muy insatisfecho	366 (56,7)	280 (43,3)	0,013
	Insatisfecho	651 (53,4)	567 (46,6)	
	Ni satisfecho, ni insatisfecho	1.260 (51,6)	1.184 (48,4)	
	Satisfecho	1.313 (50,5)	1.289 (49,5)	
	Muy satisfecho	1.507 (54,0)	1.283 (46,0)	
Nivel de satisfacción con relaciones familiares	Muy insatisfecho	71 (57,7)	52 (42,3)	0,017
	Insatisfecho	161 (61,9)	99 (38,1)	
	Ni satisfecho, ni insatisfecho	562 (52,9)	500 (47,1)	
	Satisfecho	1.427 (51,4)	1.349 (48,6)	
	Muy satisfecho	2.876 (52,5)	2.603 (47,5)	
Nivel de satisfacción con la democracia	Muy insatisfecho	500 (47,9)	544 (52,1)	0,023
	Insatisfecho	1.002 (53,8)	862 (46,2)	
	Ni satisfecho, ni insatisfecho	2.393 (52,5)	2.161 (47,5)	
	Satisfecho	975 (53,7)	841 (46,3)	
	Muy satisfecho	227 (53,8)	195 (46,2)	
Visualización de la percepción de Chile en 5 años	Mejor que ahora	1.856 (52,7)	1.667 (47,3)	0,570
	Igual que ahora	1.833 (51,9)	1.699 (48,1)	
	Peor que ahora	1.408 (53,2)	1.237 (46,8)	

Fuente: Elaboración propia, a partir de Décima Encuesta Nacional de Juventudes 2022^(13)^.

Bajo el supuesto de normalidad, se pueden identificar significativas asociaciones entre las características de la población estudiada, con la participación política activa en los últimos 12 meses. Destaca sexo femenino como principal participante político, respecto al masculino, con significancia considerable (valor de p = 0,00). Quienes autorreportan niveles de escolaridad medio refieren participación política importante (valor de p = 0,00) en otros tipos de escolaridad alcanzados en el grupo etario. PJ solteras (valor de p = 0,00), no jefes de hogar (valor de p = 0,00), autodefinidas como felices (valor de p = 0,053), con una mirada positiva de su propia vida en futuro (valor de p = 0,00), satisfechas con economía actual (valor de p = 0,051), con salud (valor de p = 0,00), con tiempo de descanso (valor de p = 0,013), con tiempo disponible para compartir con amigos y familia (valor de p = 0,017) y con democracia del país (valor de p = 0,023) demostraron asociaciones significativas. Y PJ sin identificación alguna en sectores políticos determinados (valor de p = 0,00) ni con intereses en materias políticas (valor de p = 0,00) se autodefinen como no participantes activos en política, con asociaciones significativas.

## DISCUSIÓN

El presente proceso investigativo permitió identificar ciertas asociaciones entre factores sociodemográficos y aspectos relacionados con la vida y la salud y la PC del PJ en Chile. Resulta significativo pues comprender las expectativas de participación comunitaria relacionadas con la edad puede ayudar a los formuladores de políticas en el diseño y adaptación de servicios apropiados.

Los resultados muestran un nivel de interés en política del 27,1%, lo cual es positivo al considerar que cuando las personas se involucran como representantes públicos, logran plantear preocupaciones colectivas^(7,10)^ sobre materias diversas, en donde la salud supone gran interés. Esto lo demuestra el hecho de que el 88,9% ve su vida mejorando en cinco años. En el mismo contexto, se plantea que la PC es precisa para lograr una distribución del poder político, lograr un poder comunitario para impactar en PP y tener un cambio social^(15)^. Desarrollan una escalera de PC, donde con implementación de mecanismos de control comunitario a largo plazo e inversión de recursos, la ciudadanía será capaz de una PC. Asimismo, introduce la idea de financiamiento de privados para este tipo de procesos. Esto es crítico pues el nivel de satisfacción con la democracia que caracteriza el país tiene una asociación significativa con la participación activa en los últimos 12 meses.

Destaca el nivel de satisfacción con la salud con un 78,2% muy satisfecho o satisfecho en el contexto de COVID-19, donde se vivió un periodo de confinamiento en el país, mostrando un 52% con muy buena o buena percepción de los servicios de salud^(10)^. Un estudio en Argentina plantea que la modalidad no presencial propició tranquilidad en la preservación de la salud de las personas^(10)^.

Es complejo, ya que casi la mitad de las personas (46,9%) no suele responder sobre su nivel de satisfacción con la democracia. Según un estudio realizado en Canadá, la gente necesita que las políticas sanitarias consideren las opiniones públicas^(16)^. Así, en Colombia el 89% aprobaría la gestión gubernamental si desarrollara políticas de cohesión social^(17)^.

En adición, la literatura enfatiza en que se espera una mayor participación pública de personas en procesos políticos, pues el involucrarse podría fomentar un acuerdo sobre qué las prioridades en la toma de decisiones. Los resultados muestran solo un 5,8% de PJ que desean participar en la elaboración de PP para la juventud, en concordancia con una investigación en Colombia que menciona una antipatía frente a temas de política^(17)^. Los bajos niveles de participación política en los últimos 12 meses pudieron verse relacionados con el confinamiento del COVID-19, debido a la propia situación de aislamiento y la falta de priorización de necesidades al respecto. Este proceso pandémico permite relacionarse con un aumento de la participación en ayuda a la comunidad o voluntariado en los últimos 12 meses, en comparación con el resto de las participaciones, con un incremento de participación en juntas vecinales, comedores de barrio, entre otros^(10)^.

El 56,5% de los participantes de esta encuesta se encuentra muy satisfecho con el nivel de satisfacción con sus relaciones familiares, según un estudio realizado en Colombia que demuestra que la mayor interacción de las personas es con su familia (77,3%). Además, es el grupo con mayor relación (88,6%). A nivel colectivo, el 73,5% declara interés en participar en sus localidades^(17)^. Este punto es fundamental, ya que las investigaciones realizadas en el Reino Unido y Sudáfrica destacan la importancia del beneficio mutuo para lograr la PC. En equipos donde no había una visión de trabajo colaborativo, con decisiones dependiendo de una sola persona, el equipo se desintegró sin lograr participación. En el primero, destaca una PC por medio de investigación con fondos públicos, y en el segundo, se enfatiza un enfoque de justicia social con resultados útiles a la comunidad^(1)^.

Esta investigación mostró asociación significativa de la PC con el nivel de satisfacción con la salud, así como un estudio es Suecia, donde la participación es impactada por la buena atención de salud^(2)^.

La promoción de salud y prevención de enfermedades resulta significativa cuando se pretende potenciar desde la educación en salud, pues con poblaciones educadas es posible fortalecer la PC en las decisiones de cuidado^(18)^. Esto se respalda en que el 89% de la población le gustaría capacitarse en temas relacionados con política, así como participar en reuniones con autoridades gubernamentales, con el fin de proponer soluciones y no sólo obtener información sobre los problemas que les aquejan^(17)^.

Igualmente, esta pesquisa respalda que la participación política activa en los últimos 12 meses tiene asociación significativa con la falta de identificación en sectores políticos, intereses en la materia o no autodefinirse como participante activo en política. En ello, las estrategias comunicativas, como las campañas de comunicación enmarcadas en un equilibrio entre efectividad y ética^(19)^, representan enfoques de salud pública.

En este contexto, en Chile, las tensiones socio políticas y sanitarias recientes debido a la pandemia de COVID-19 pudieron haber generado cierto estrés y fatiga entre la ciudadanía^(20)^. Sin embargo, estrategias de PC como promover espacios de diálogo constructivo permiten abordar diferencias de manera productiva y llegar a acuerdos que sean aceptables para la mayoría de la comunidad^(21,22)^.

Las PJ presentan asociación significativa entre tiempo disponible para compartir con amigos y familia y la PC; así, la PC puede impactar en promoción y prevención de salud. Un estudio en México sostiene que lazos sociales cercanos y la familia logran potenciar y fortalecer la cohesión-participación del individuo con su entorno^(17)^, lo que resulta en conductas biopsicosociales más saludables^(23–25)^. En PJ, resulta significativo, ya que el desarrollo, de una perspectiva sustancial de ciudadanía, puede favorecer la incorporación de los jóvenes en la vida pública siempre que se construya un contexto social, cultural e institucional basado en la participación significativa y en el diálogo con la juventud^(26)^.

El nivel de satisfacción con la democracia se asocia significativamente con la participación política los últimos doce meses. En Colombia, un 56% de los entrevistados evalúa al gobierno con un 6/10^(17)^. Apropiarse del derecho a participar se reconoce como una de las prácticas esenciales para ser un ciudadano activo, donde previamente deben estar informados y presentar pertenencia a la comunidad^(26)^. Así, el confinamiento del COVID-19 permitió percibir de otra forma el ambiente, creando mayores vínculos con el vecindario^(10)^. De este modo, se debe tener presente que la percepción que tienen las personas de su sentido de pertenencia social con la familia y lugar de origen impacta en su desarrollo cívico electoral^(17)^.

Se reconoce que el primer paso para ser un ciudadano activo es estar informado^(26)^, pero para lograr niveles más altos de PC también es necesario informar y escuchar a las partes interesadas y utilizar la retroalimentación de las personas en la toma de decisiones. Igualmente, este estudio en EEUU sugiere que la PC es una fuente de mejora frente a las violaciones regulatorias, y sugiere que la PC puede impactar positivamente en la administración, calidad de la atención, derechos de las personas, entre otros, destacando el valor de la participación en áreas como la salud, altamente profesionalizados, donde existen gran cantidad de normativas y capacitación^(3)^.

Asimismo, se destaca la necesidad de participación de las personas en las políticas que orientan los servicios sociales, aunque el camino para implementarlas en las PP sea dificultoso. Los enfoques del personal respecto de la participación de los usuarios son cruciales para las oportunidades de las personas de participar en la toma de decisiones, en particular en el caso de aquellas con necesidades de cuidados extensas y autonomía reducida^(2)^.

La PC posee trascendencia en modelos políticos^(22)^, pues enfatiza las necesidades reales de las personas, quienes, en alianza con otros actores, pueden incidir en la agenda y ser partícipes de sus propias soluciones, siendo imprescindible que el gobierno se constituya en moderador en la toma de decisiones y controle el poder político^(27–29)^. Así, PC y cultura generan impacto social contundente^(26)^.

En complemento, las investigaciones en PC son claves para la coproducción y desarrollo de las PP^(3,4)^. A medida que aumentan las oportunidades de participación pública en la investigación en salud, se anticipa una mayor participación^(16)^. En tal sentido, se debe considerar que la participación pública es una exigencia para obtener financiamiento en salud en diversas instituciones^(30)^.

Finalmente, el concepto de PC se ha visto impactado por presiones de ser utilizado como símbolo de grupos, que colocan PC inclusiva y diversa como una prioridad^(4)^. Como limitaciones, este estudio muestra solamente una versión de la encuesta. Se sugiere en otras investigaciones comparar con periodos no pandémicos para reconocer la secuencia de evolución en el tiempo de la PC en PJ.

## CONCLUSIÓN

El desarrollo de la presente pesquisa permitió analizar la asociación de factores sociodemográficos, aspectos relacionados con la vida y la salud sobre la PC de PJ en Chile. El sexo femenino describe mayor participación política, y niveles de escolaridad refieren participación política importante. PJ solteras, no jefes de hogar, autodefinidos como felices, con mirada positiva de su propia vida en el futuro, satisfechos con economía actual, salud, tiempo de descanso y disponible para compartir con amigos y familia y con la democracia que caracteriza el país demostraron asociaciones significativas. Asimismo, las PJ sin identificación alguna en sectores políticos determinados, ni con intereses en materias políticas, se autodefinen como no participantes activos en política, con asociaciones significativas. Las PJ son fundamentales para construir ciudadanía futura. En ello, las características que le son propias deben fortalecerse mediante estrategias de PC efectivas, mediadas por educación que le permitan ser conscientes de su propio valor como personas que impactan en PP.

## Data Availability

Todo el conjunto de datos que respalda los resultados de este estudio están a disposición en el Centro de Documentación Digital Instituto Nacional de la Juventud Gobierno de Chile con el URL https://www.injuv.gob.cl/encuestanacionaldejuventud en noviembre de 2022.
